# Ki-67 is strongly prognostic in synovial sarcoma: analysis based on 86 patients from the Scandinavian Sarcoma Group Register

**DOI:** 10.1038/sj.bjc.6690602

**Published:** 1999-08

**Authors:** B T Skytting, H C Bauer, R Perfekt, G Nilsson, O Larsson

**Affiliations:** Department of Orthopedics, Stockholm Soder Hospital, Stockholm, SE-100 64, Sweden; Department of Orthopedics and Oncology Service and Cellular and Molecular Tumour Pathology, Karolinska Hospital, Stockholm, SE-171 76, Sweden; Southern Swedish Regional Tumour Registry, Lund University Hospital, Lund, SE-221 85, Sweden

**Keywords:** synovial sarcoma, Ki-67, multivariate analysis, prognosis

## Abstract

In a study based on formalin-fixed paraffin-embedded material from 86 patients with primary synovial sarcoma located in the extremities or on the trunk wall, the prognostic importance of MIB-1 index, p53-expession and tumour size was analysed. Multivariate analysis identified two metastatic risk factors: increasing tumour size and MIB-1 >9%. The 5-year metastasis-free survival-rate for patients with tumour size ≤5 cm + MIB-1 <10% was 0.83 (95% confidence interval (CI) 0.64–0.92) compared to 0.31 (95% CI 0.11–0.53) in cases with tumour size >5 cm + MIB-1 ≥10%. Our study shows that metastatic disease in synovial sarcoma is closely related to MIB-1 index. Using our model based on tumour size and MIB-1 index, cases with good and poor prognosis can easily be discriminated. Therefore our model can be used to identify patients who should be considered for adjuvant chemotherapy. © 1999 Cancer Research Campaign


*British Journal of Cancer* (1999) **80**(11), 1809–1814
© 1999 Cancer Research Campaign
Article no. bjoc.1999.0602

### Ki-67 is strongly prognostic in synovial sarcoma: analysis based on 86 patients from the Scandinavian Sarcoma Group Register

**BT Skytting[1], HC Bauer[2], R Perfekt[4], G Nilsson[3] and O Larsson[3]**

[1]Department of Orthopedics, Stockholm Soder Hospital, SE-100 64 Stockholm, Sweden; [2]Oncology Service, Department of Orthopedics and [3]Cellular and Molecular Tumour Pathology, Karolinska Hospital, SE-171 76 Stockholm, Sweden; [4]Southern Swedish Regional Tumour Registry, Lund University Hospital, SE-221 85 Lund, Sweden

**Summary** In a study based on formalin-fixed paraffin-embedded material from 86 patients with primary synovial sarcoma located in the extremities or on the trunk wall, the prognostic importance of MIB-1 index, p53-expession and tumour size was analysed. Multivariate analysis identified two metastatic risk factors: increasing tumour size and MIB-1 >9%. The 5-year metastasis-free survival-rate for patients with tumour size ≤5 cm + MIB-1 <10% was 0.83 (95% confidence interval (CI) 0.64–0.92) compared to 0.31 (95% CI 0.11–0.53) in cases with tumour size >5 cm + MIB-1 ≥10%. Our study shows that metastatic disease in synovial sarcoma is closely related to MIB-1 index. Using our model based on tumour size and MIB-1 index, cases with good and poor prognosis can easily be discriminated. Therefore our model can be used to identify patients who should be considered for adjuvant chemotherapy.

**Keywords:** synovial sarcoma; Ki-67; multivariate analysis; prognosis

Synovial sarcoma accounts for 5–10% (Choong et al, 1994) of all soft tissue sarcomas and occurs most often in adolescents and young adults (Cadman et al, 1965). A tumour-specific translocation t(X;18) (p11.2;q11.2) (SYT/SSX) (Limon et al, 1991), is represented in more than 95% of the cases. Almost all synovial sarcomas are histopathologically classified high-grade lesions. The reported 5-year overall survival rate varies from 40 to 70% (Wright et al, 1982; Choong et al, 1995). Besides tumour size, which has been shown to be associated with poor clinical outcome (Hajdu et al, 1977; Brodsky et al, 1992; Skytting et al, submitted), there are few objective markers predicting the prognosis of this malignant disease.

The p53 gene, a tumour suppressor gene, is believed to play an active role in the cell growth control (Raycroft et al, 1990) by acting as a regulatory checkpoint in the cell cycle, arresting the cells in G1 phase if DNA damage has occurred (Yin et al, 1992). In contrast, mutated p53 fails to block cell cycle progression (Nigro et al, 1989). Therefore, p53 mutations may contribute to uncontrolled cell growth. Point mutation of mis-sense type produce a product with a considerable longer half-life compared to germline p53, leading to accumulation of defect p53 protein in the nucleus, that can be assayed by immunohistochemistry (Finlay et al, 1988). However, the significance of p53 immunostaining is controversial since false negative detection due to e.g. nonsense-, frame-shift-, splize mutations, or gross deletions (Wadayama et al, 1993) and false positive detection due to stabilization of wild-type p53 by e.g. Mdm2 or DNA damage in cells (Cordon-Cardo et al, 1994), exists. Nevertheless, association between positive p53 immunostaining and impaired clinical course has been reported (Drobnjak et al, 1994; Kawai et al, 1994; Wurl et al, 1997).

Ki-67 is an antigen exclusively expressed during the proliferating phase of the cell cycle (Gerdes et al, 1984). The function of Ki-67 is still unknown, but it is a well established marker for proliferation. Since MIB-1 index recognizes all phases of the cell cycle (not in G0) it is a considerably more sensitive proliferation marker than mitotic index.

The prognostic significance in soft tissue sarcomas (STS) regarding both these markers remains unclear, mainly due to small or heterogeneous materials. Patients with high/low tumour grades, primary/recurrent tumours, various tumour sites (retroperitoneal mixed with extremities) and different soft tissue entities have been included in the same materials with a variety of results. In a recent study concerning primary high-grade mixed STS, Ki-67 was shown to be an independent prognostic marker (Heslin et al, 1998) but in a material with malignant fibrous histiocytomas it was not (Zehr et al, 1990). In a large material of mixed STS, over-expression of p53 was associated with reduced survival (Drobnjak et al, 1994) but in another large study of mixed STS this was not confirmed (Nakanishi et al, 1997). So far neither MIB-1 nor p53 has been assessed for a large number of synovial sarcoma patients.

The purpose of this study was to analyse the prognostic importance of proliferation index, measured by the MIB-1 monoclonal antibody, and the expression of p53 in a large material of clinically and histopathologically well-characterized synovial sarcoma patients.

*Received 21 October 1998*
*Revised 28 January 1999*
*Accepted 4 February 1999*

*Correspondence to:* O Larsson

### MATERIALS AND METHODS

### Patients

The study was based on 104 patients diagnosed with synovial sarcoma of the extremities or trunk wall between 1986 and 1994 (Skytting et al, submitted). Eighteen patients were excluded because tumour material was not available, leaving 86 patients for study.

The histopathological criteria for synovial sarcoma were those of Enzinger and Weiss (Enzinger and Weiss, 1995). The SSG-pathology-board, a selected group of pathologists from the participating centres, re-examined all available original slides from the primary tumours without knowledge of the clinical course. In selected cases new slides, immunohistochemical stainings or RT-PCR (for detection of SYT-SSX hybrid products) were performed for final diagnosis.

Medical records were reviewed in all cases to verify and complete reported clinical data. There were 47 males and 39 females with a median age of 39 (6–81) years. Eight tumours were located on the trunk wall, 41 in the proximal part of the extremities (shoulder, upper arm, elbow, groin, gluteus, thigh and knee) and 37 in the distal part (lower arm, hand, lower leg and foot). The median tumour size was 5 (1–20) cm. All tumours were high-grade lesions (Grade III and Grade IV) on a 4-grade scale (Broders and Hargrave, 1939; Angervall et al, 1986) except for one (Grade II). In 34 patients, the final surgical margin was intralesional or marginal, and in 52 wide or compartmental. None had preoperative radiotherapy but 20 were treated postoperatively due to an intralesional or marginal surgical margin. Four patients, three children and one young adult, had adjuvant chemotherapy for primary tumour. There was no systemic bias regarding post-operative treatment and these markers.

No patient was lost to follow-up. The median follow-up for survivors ($n$ = 53) was 6 years (2–11) years. Thirty-one patients (36%) developed metastases at a median of 1.5 years. The most common site was the lungs.

### Immunohistochemical examinations

All original haematoxylin and eosin-stained slides were re-examined and matched to the corresponding paraffin-embedded tissue blocks. One representative block per tumour was selected for immunostaining. Immunostaining was performed according to the standard ABC-technique (Elite Standard Kit. Cat. PK-6100; Vector, Burlingame, CA, USA). Paraffin sections were deparaffinized, rehydrated and pretreated. Antigen retrieval was performed by immersing the specimens for 10 min in a citrate buffer at pH 6 and heating in a microwave oven (700 W) for 10 min. After rinsing, the endogenous peroxidase activity was blocked by hydrogen peroxide dissolved in methanol (3% hydrogen peroxide to methanol, 1:5 by volume) for 30 min. The

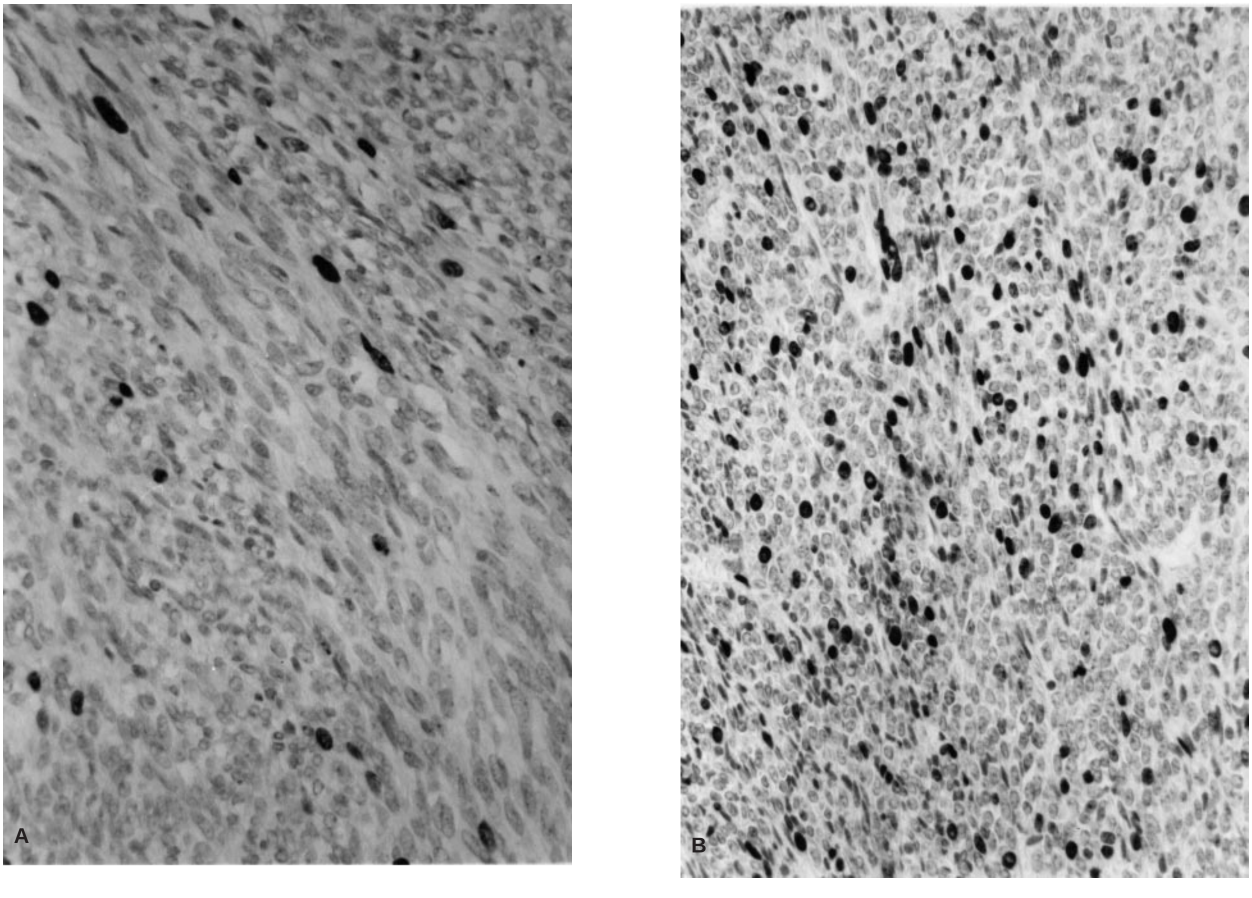

**Figure 1** Photomicrographs of two synovial sarcoma cases studied using immunohistochemical staining with MIB1 antibody. Low proliferating activity: MIB-1 index = 0–9% (**A**). High proliferating activity: MIB-1 index >9% (**B**)

*© 1999 Cancer Research Campaign*

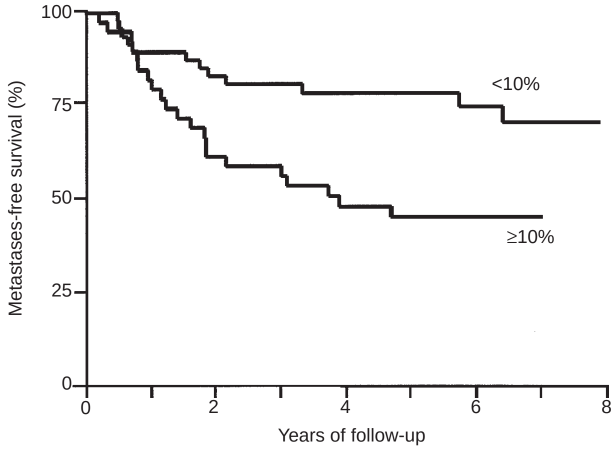

**Figure 2** Kaplan–Meier estimates for metastasis-free survival among 84 synovial sarcoma patients based on low (0–9%) *n* = 46 and high (>9%) *n* = 38 proliferative MIB-1 index, *P* = 0.007 (log-rank test)

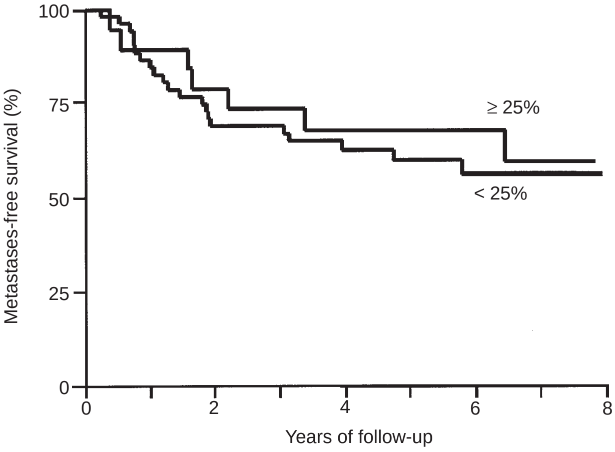

**Figure 4** Kaplan–Meier estimates for metastasis-free survival among 70 synovial sarcoma patients based on low (0–24%) *n* = 51 and high (>24%) *n* = 19 p53, *P* = 0.007 (log rank test)

sections were then rinsed and incubated with blocking serum (normal horse serum) for 20 min and later incubated with primary antibodies: anti-Ki-67 (MIB-1; Immunotech, Marseille, France) 1:50 and anti P-53 (DO-1, SDS; Santa Cruz Biotechnology, Santa Cruz, CA, USA) 1:100. All incubations were performed overnight at 8°C. Following the ABC-complex, a biotinylated antimouse immunoglobulin G was used as a secondary antibody. The peroxidase reaction was developed using 3,3-diaminobenzidine (diaminobenzidine tetrahydrochloride, 0.6 mg $ml^{-1}$ with 0.03% hydrogen peroxide) for 6 min. Haematoxylin was used as the nuclear counterstain. Tris–phosphate-buffered saline (pH 7.6) was used for rinsing between the steps. The staining was checked with negative and positive controls.

A semiquantitative score was employed to assess the percentage of cells that were positively stained regardless of staining intensity. The percentage of MIB-1- and p53-positive cells per 10 high power field (× 250) were graded as follows: 0–1%, 2–9%, 10–24%, 25–49%, 50–74% and 75–100%.

All the immunohistochemically stained slides (which were coded) were analysed microscopically independently by BTS and OL without knowledge of the clinical characteristics. There was concurrence of assessment in 144 of 154 specimen and over- or underestimation of one score grade in ten. A consensus was reached for these ten cases. In each case more than 1000 cells were analysed.

### Statistics

Metastases-free survival was analysed multivariately according to Cox's regression techniques supplemented with univariate comparisons using the log-rank test and Kaplan–Meier survival estimates (Kalbfleisch and Prentice, 1980).

### RESULTS

### MIB-1 and p53

Proliferation index was assessed by the MIB-1 antibody in 84 of 86 patients. Nuclear over-expression of p53 was determined in 70 of 86 patients. All of the material could not be stained for both MIB-1 and p53 due to lack of tumour specimen. The distribution of MIB-1-labelled nuclei among the 84 cases was: 0–1%, 13; 2–9%, 33; 10–24%, 22; 25–49%, 14; 50–74%, 1; 75–100%, 1. A MIB-1 index of 10% or more was considered *highly proliferative* (Figure 1). The p53 distribution was: 0–1%, 41; 2–9%, 6; 10–24%, 4; 25–49%, 11; 50–74%, 8. Specimens were at least 25% of the nuclei stained positive for p53 were regarded as carrying p53 mutations in close conformity with other studies (Drobnjak et al, 1994).

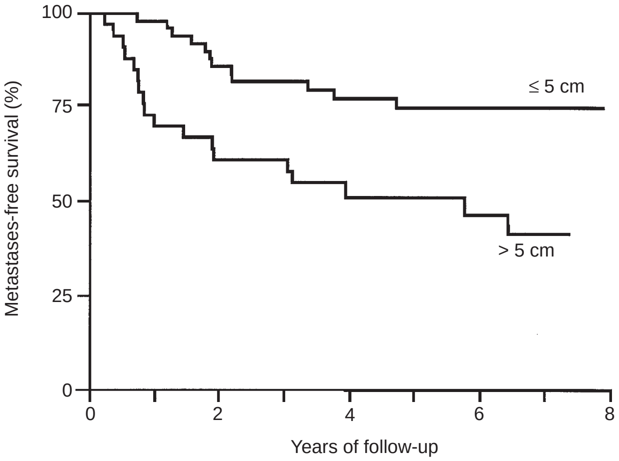

**Figure 3** Kaplan–Meier estimates for metastasis-free survival among 82 synovial sarcoma patients based tumour size ≤5 cm *n* = 49 and tumour size >5 cm *n* = 33, *P* = 0.003 (log-rank test) (tumour size missing in four patients)

**Table 1** Metastasis-free survival among 80 synovial sarcoma patients with respect to MIB-1 index and tumour size according to Cox's proportional hazard model

| Criteria | Hazard ratio | 95% Confidence interval | *P*-value |
|---|---|---|---|
| Tumour size 1–3 cm | (Reference) | – | – |
| Tumour size 4–5 cm | 3.9 | 0.86–18 | 0.08 |
| Tumour size 6–20 cm | 7.1 | 1.6–31 | 0.009 |
| MIB-1 ≥10% | 2.2 | 1.0–4.6 | 0.04 |

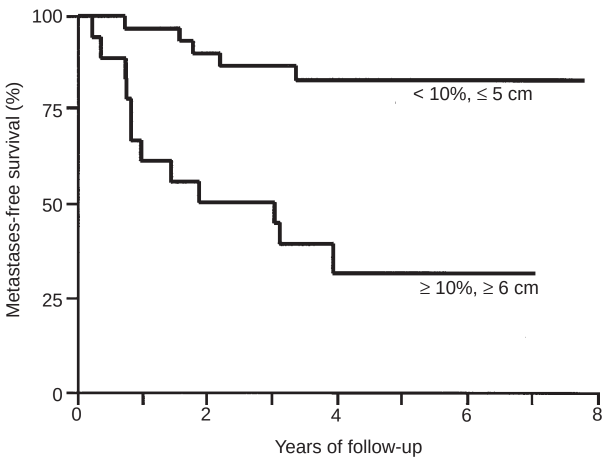

**Figure 5** Kaplan–Meier estimates for metastasis-free survival in a low-risk group $n$ = 30 (tumour size ≤5 cm and MIB-1 <10) versus a high-risk group $n$ = 18 (tumour size >5 and MIB-1 ≥10), $P$ = 0.007 (log-rank test)

In 38 out of 84 patients the MIB-1 index was ≥10%. The staining pattern was generally evenly distributed throughout the specimen and a clear-cut labelling of the tumour cells was achieved. However, three slides in the *low-proliferative* group stained unevenly, suggesting two or more cell-clones in the same specimen. Two out of three of these patients developed metastases (data not shown). There was a significant difference in the metastasis-free survival rate for patients with a MIB-1 index ≥10% versus patients with a low index (log-rank *P*-value = 0.007) (Figure 2). Twenty-one out of 38 patients developed metastases in the highly proliferative group and only 12 out of 46 in the low-proliferative group. A significant difference regarding metastasis-free survival for patients with tumour size ≤5 cm and >5 cm was also obvious (log-rank *P*-value = 0.003) (Figure 3).

No significant difference in metastasis-free survival was detected for patients staining highly positive for p53 versus those who did not (log-rank *P*-value = 0.63) (Figure 4). Seven out of 19 patients developed metastases in the group where p53 was over expressed versus 19 of 51 where it was not.

### Multivariate analysis

Tumour size has been introduced as a strong prognostic marker for metastasis-free survival in synovial sarcoma (Choong et al, 1994; Skytting et al, submitted). Tumour size was available for all but four patients. We decided to recode tumour size as a categorical covariate with three levels: 1–3, 4–5 and 6–20 cm respectively. The categorization was used since the log hazard did not appear to increase linearly with tumour size. Both tumour size (1–3, 4–5, 6–20 cm) and MIB-1 index were entered in a multivariate Cox regression analysis for metastasis-free survival (Table 1). Although larger tumours were slightly more likely to have MIB-1 index >9% both factors gave a significant contribution to the model.

Figure 5 illustrates metastasis-free survival probabilities for patients with tumour size at most 5 cm and MIB-1 less than 10% compared to patients with tumour size more than 5 cm and MIB-1 above 9%. As shown, 5-year survival rate among the 30 patients with small and low-proliferative tumours was as high as 0.83 (95% CI 0.64–0.92), whereas it was only 0.31 (95% CI 0.11–0.53) among the 18 with large and highly proliferative synovial sarcomas.

### DISCUSSION

Our study shows that MIB-1 index and tumour size are strongly related to metastasis-free survival in primary synovial sarcomas. Prognosis for synovial sarcomas and other STSs has so far been based on a clinical and histopathological staging systems built up by histological grade, tumour size and tumour spread. However, a majority of synovial sarcomas are considered to be high-grade lesions and in our material only one tumour was of low grade. Furthermore, lack of concrete criteria for grading, different grading systems and variable interobserver reproducibility contributes to subjective interpretations (Coindre et al, 1986). In contrast, MIB-1 index and tumour size can be assessed objectively.

The monoclonal antibody Ki-67 exclusively reacts with a protein (Ki-67) expressed only during the proliferating phase of the cell cycle (late G1, G2, S and mitosis) (Gerdes et al, 1984). In a number of publications Ki-67 has proven to be a reliable marker for measuring cell growth in human neoplasmas (Brown et al, 1990). However, Ki-67 could only be applied on fresh tissue since the antigen which it detects is denaturated by tissue fixation. With the introduction of the MIB-1 antibody, a true Ki-67 equivalent, and a new antigen retrieval technique with the use of microwaves (Shi et al, 1991), immunostaining of formalin-fixed paraffin-embedded (FFPE) material was possible with a high reproducibility, equalling results obtained in fresh material (Gerdes et al, 1992). A close correlation was found between the FFPE material stained with MIB-1 and parallel fresh tissue stained with Ki-67 (Cattoretti et al, 1992).

Previous results regarding the prognostic value of high levels of Ki-67 or MIB-1 in STS varies; in angiosarcoma an association was seen with poor prognosis (Meis-Kindblom et al, 1998) but this observation was not repeated in a material of MFH (Zehr et al, 1990). Various results have been reported from mixed series, usually non-significant in multivariate analysis except in Heslin's study of high-grade STS (Heslin et al, 1998). Since, only a few histotype-specific studies regarding the prognostic value of Ki-67 have been published and there are reasons to believe that the expression level differs from histotype, the prognostic significance of Ki-67 might differ between different STS entities.

The cut-off values for MIB-1 index varies a lot throughout the literature, probably depending on the tissue material observed and possibly by the evaluation (median value/area of greatest density of staining). For sarcomas a cut-off value in the range of 10% (Choong et al, 1995) to 40% (Levine et al, 1997) has been utilized. Following Choong and co-workers, we divided the material into two groups of approximately equal sizes, based on the employed semiquantitative *score*. A cut-off value of MIB <10% defined a group with better metastasis-free survival rates compared to the group with MIB ≥10% (log-rank *P*-value = 0.007). As indicated by our results it is possible that the presence of highly proliferating cell clones among tumours in the *low-proliferative* group may predict a worse prognosis. However, our material was too small (only three cases) to give a conclusive answer to this question.

Image analysis technology has been used for the assessment of Ki-67 staining partly to reduce intraobserver variability (Zehr et al, 1990). However, with a clear cut staining pattern for positive nuclei, typically for MIB-1, we did not experience difficulties in scoring the specimens manually.

 *© 1999 Cancer Research Campaign*

Mutations of TP-53 and alterations in p53 protein expression, has been described to occur in STS (Drobnjak et al, 1994). They found in a mixed series of 174 patients with fresh frozen materials of STS, a significantly reduced over all survival among patients with p53 nuclear over-expression >20%. However, when only high-grade lesions were taken into account, the difference was not significant. Our study, based on paraffin-embedded tissues, revealed nuclear overexpression in 19 of 70 tumours (27%) which is close to what Drobnjak reported (Drobnjak et al, 1994). However, we could not detect a difference in metastasis-free survival between patients staining for less than 25% for p53 and those who did not. Recently, the mdm2 gene responsible for producing a protein that binds to p53 and eliminates its ability to function as a transcription factor, has been shown to be amplified in STS, resulting in an inactivation of wild-type p53. High levels of Mdm2 has also been associated with poor survival especially with an over-expression of p53 in the same tumour (Cordon-Cardo et al, 1994). However, since the Mdm2 protein was not assessed in this study the clinical significance can not be evaluated. Evaluation regarding prognostic relevance in STS of different p53 antibodies revealed a positive marker frequency of 36–63% (Wurl et al, 1997). The antibody, DO-1 that we used came out on top in this study, indicating the right choice.

In conclusion, this study identifies a high-risk group of patients with MIB-1 index ≥10%, and tumour size >5 cm in primary synovial sarcoma. Prognostication with magnetic resonance imaging (MRI) for assessment of tumour size and core needle biopsy for MIB-1 index would make it possible to identify *high-risk* patients, before surgery, who can be considered for adjuvant chemotherapy, and on the contrary identify *low-risk* patients who should be managed by local treatment only. To the best of our knowledge, the present study is the first describing MIB-1 index to be a prognostic marker for metastasis-free survival in a well-defined STS entity.

### ACKNOWLEDGEMENTS

Supported by the Cancer Society in Stockholm (project nos. 97:109, 97:153), the Nordic Cancer Union, the Swedish Cancer Society, and grants from the Karolinska Institute. The following have been responsible for reporting primary patient data and follow-up:

Henrik CF Bauer, Oncology Service, Department of Orthopedics, Karolinska Hospital, Stockholm, Sweden.
Örjan Berlin, Department of Orthopedics, Sahlgrenska Hospital, Gothenburg, Sweden.
Per Gustafson, Department of Orthopedics, Lund University Hospital, Lund, Sweden.
Carl Blomqvist and Riika Huuhtanen, Department of Radiotherapy and Oncology, Helsinki University Hospital, Helsinki, Finland.
Ragnhild Klepp, Department of Oncology, Regional Hospital, Trondheim, Norway.
Richard Löfvenberg, Department of Orthopedics, Umeå University Hospital, Umeå, Sweden.
Gunnar Sæter, Department of Oncology, The Norwegian Radium Hospital, Oslo, Norway.
Clement S. Trovik, Department of Orthopedics, Haukeland University Hospital, Bergen, Norway.
Ola Wahlström, Department of Orthopedics, University Hospital, Linköping, Sweden.

### REFERENCES

Angervall L, Kindblom L-G, Rydholm A and Stener B (1986) The diagnosis and prognosis of soft tissue tumors. *Semin Diagn Pathol* **3**: 240–258

Broders AC and Hargrave R (1939) Pathologic features of soft tissue fibrosarcoma. *Surg Gynecol Obstet* **69**: 267–280

Brodsky JT, Burt ME, Hajdu SI, Casper ES and Brennan MF (1992) Tendosynovial sarcoma. Clinicopathologic features, treatment, and prognosis. *Cancer* **70**: 484–489

Brown DC and Gatter KC (1990) Monoclonal antibody Ki-67: its use in histopathology. *Histopathology* **17**: 489–503

Cadman NL, Soule EH and Kelly PJ (1965) Synovial sarcoma. *Cancer* **18**: 613–623

Cattoretti G, Becker MHG, Key G, Duchrow M, Schluter C, Galle J and Gerdes J (1992) Monoclonal antibodies against recombinant parts of the Ki-67 antigen (MIB1 and MIB3) detect proliferating cells in microwave-processed formalin-fixed paraffin sections. *J Pathol* **168**: 357–363

Choong PFM, Åkerman M, Willen H, Andersson C, Gustafson P, Baldetorp B, Fernö M, Alvegård T and Rydholm A (1994) Prognostic value of Ki-67 expression in 182 soft issue sarcomas. Proliferation – a marker of metastasis? *APMIS* **102**: 915–924

Choong PFM, Pritchard DJ, Sim FH, Rock MG and Nascimento AG (1995) Long-term survival in high grade soft tissue sarcoma: prognostic factors in synovial sarcoma. *Int J Oncol* **7**: 161–169

Cordon-Cardo C, Latres E, Drobnjak M, Oliva MR, Pollak D, Woodruff JM, Marechal V, Chen J, Brennan MF and Levine AJ (1994) Molecular abnormalities of mdm2 and p53 genes in adult soft tissue sarcomas. *Cancer Res* **54**: 794–799

Coindre JM, Trojani M, Contesso G, David M, Rouesse J, Bui NB, Bodaert A, De Mascarel I, De Mascarel A and Goussot JF (1986) Reproducibility of a histopathologic grading system for adult soft tissue sarcoma. *Cancer* **58**: 306–309

Drobnjak M, Latres E, Pollak D, Karpeh M, Dudas M, Woodruff JM, Brennan MF and Cordon-Cardo C (1994) Prognostic implications of p53 nuclear overexpression and high proliferation index of Ki-67 in adult soft-tissue sarcomas. *J Natl Cancer Inst* **86**: 549–554

Enzinger FM and Weiss SW (1995) *Soft Tissue Tumours* Mosby: St Louis

Finlay CA, Hinds PW, Tan TH, Eliyahu D, Oren M and Levine AJ (1988) Activating mutations for transformation by p53 produce a gene product that forms an hsc70-p53 complex with an altered half-life. *Mol Cell Biol* **8**: 531–539

Gerdes J, Lemke H, Baisch H, Wacker HH, Schwab U and Stein H (1984) Cell cycle analysis of a cell proliferation-associated human nuclear antigen defined by the monoclonal antibody Ki-67. *J Immunol* **133**: 1710–1715

Gerdes J, Becker MHG and Key G (1992) Immunohistochemical detection of tumour growth fraction (Ki-67 antigen) in formalin-fixed and routinely processed tissues. *J Pathol* **168**: 85–87

Hajdu SI, Shui MH and Fortner JG (1977) Tendosynovial sarcoma. *Cancer* **39**: 1201–1217

Heslin MJ, Cordon-Cardo C, Lewis JJ, Woodruff JM and Brennan MF (1998) Ki-67 detected by MIB-1 predicts distant metastasis and tumour mortality in primary, high grade extremity soft tissue sarcoma. *Cancer* **83**: 490–497

Kalbfleisch JD and Prentice RL (1980) *The Stistical Analysis of Failure Time Data*. Wiley: New York

Kawai A, Noguchi M, Beppu Y, Yokoyama R, Mukai K, Hirohashi S, Inoue H and Fukuma H (1994) Nuclear immunoreaction of p53 protein in soft tissue sarcomas. *Cancer* **73**: 2499–2505

Levine EA, Holzmayer T, Bacus S, Mechetner E, Mera R, Bolliger C, Roninson IB and Das Gupka TK (1997) Evaluation of newer prognostic markers for adult soft tissue sarcoma. *J Clin Oncol* **15**: 3249–3257

Limon J, Mrozek K, Mandahl N, Nedoszytko B, Verhest A and Rys J (1991) Cytogenetics of synovial sarcoma: presentation of ten new cases and review of the literature. *Genes Chromosomes Cancer* **3**: 338–345

Meis-Kindblom JM and Kindblom L-G (1998) Angiosarcoma in soft tissue: a study of 80 cases. *Am J Surg Pathol* **6**: 683–697

Nakanishi H, Ohsawa M, Naka N, Uchida A, Ochi T and Aozasa K (1997) Immunohistochemical detection of bcl-2 and p53 proteins and apoptosis in soft tissue sarcoma: their correlations with prognosis. *Oncology* **54**: 238–244

Nigro JM, Baker SJ, Preisinger AC, Jessup JM, Hostetter R, Cleary K, Bigner SH, Davidson N, Baylin S and Devilee P (1986) Mutations in the p53 gene occur in diverse human tumour types. *Nature* **342**: 705–708

Raycroft L, Wu HY and Lozano G (1990) Transcriptional activation by wild-type but not transforming mutants of the p53 anti-oncogene. *Science* **249**: 1049–1051

Shi R-R, Key ME and Kalra KL (1991) Antigen retrieval in formalin-fixed, paraffin-embedded tissues an enhancement method for immunohistochemical staining

© 1999 Cancer Research Campaign based on microwave oven heating of tissue sections. *Histochem J* **39**: 741–748
Wadayama B, Toguchida J, Yamaguchi T, Sasaki MS, Kotoura Y and Yamamuro T (1993) P53 expression and its relationship to DNA alterations in bone and soft tissue sarcomas. *Br J Cancer* **68**: 1134–1139
Wright PH, Sim FH, Soule EH and Taylor WF (1982) Synovial sarcoma. *J Bone Joint Surg* **64-A**: 112–122
Wurl P, Taubert H, Meye A, Berger D, Lautenschläger C, Holzhausen H-J, Schmidt H, Kalthoff H, Rath F-W and Dralle H (1987) Prognostic value of immunohistochemistry for p53 in primary soft tissue sarcomas: a multivariate analysis of five antibodies. *J Cancer Res Oncol* **123**: 502–508
Yin Y, Tainsky MA, Bischoff FZ, Strong LC and Wahl GM (1992) Wild-type p53 restores cell cycle control and inhibits gene amplification in cells with mutant p53 alleles. *Cell* **70**: 937–948
Zehr RJ, Bauer TW, Marks KE and Weltevreden A (1990) Ki-67 and grading of malignant fibrous histocytomas. *Cancer* **66**: 1984–1990

 *© 1999 Cancer Research Campaign*
